# Testing the precision and sensitivity of density estimates obtained with a camera‐trap method revealed limitations and opportunities

**DOI:** 10.1002/ece3.7619

**Published:** 2021-05-07

**Authors:** Pascal Pettigrew, Daniel Sigouin, Martin‐Hugues St‐Laurent

**Affiliations:** ^1^ Département de Biologie Chimie et Géographie Centre for Forest Research Université du Québec à Rimouski Rimouski QC Canada; ^2^ Forillon National Park Gaspé QC Canada; ^3^ Département de Biologie Chimie et Géographie Centre for Northern Studies Centre for Forest Research Université du Québec à Rimouski Rimouski QC Canada

**Keywords:** black bear, camera trap, density estimation, random encounter model, spatial capture–recapture

## Abstract

The use of camera traps in ecology helps affordably address questions about the distribution and density of cryptic and mobile species. The random encounter model (REM) is a camera‐trap method that has been developed to estimate population densities using unmarked individuals. However, few studies have evaluated its reliability in the field, especially considering that this method relies on parameters obtained from collared animals (*i.e*., average speed, in km/h), which can be difficult to acquire at low cost and effort. Our objectives were to (1) assess the reliability of this camera‐trap method and (2) evaluate the influence of parameters coming from different populations on density estimates. We estimated a reference density of black bears (*Ursus americanus*) in Forillon National Park (Québec, Canada) using a spatial capture–recapture estimator based on hair‐snag stations. We calculated average speed using telemetry data acquired from four different bear populations located outside our study area and estimated densities using the REM. The reference density, determined with a Bayesian spatial capture–recapture model, was 2.87 individuals/10km^2^ [95% CI: 2.41–3.45], which was slightly lower (although not significatively different) than the different densities estimated using REM (ranging from 4.06–5.38 bears/10km^2^ depending on the average speed value used). Average speed values obtained from different populations had minor impacts on REM estimates when the difference in average speed between populations was low. Bias in speed values for slow‐moving species had more influence on REM density estimates than for fast‐moving species. We pointed out that a potential overestimation of density occurs when average speed is underestimated, that is, using GPS telemetry locations with large fix‐rate intervals. Our study suggests that REM could be an affordable alternative to conventional spatial capture–recapture, but highlights the need for further research to control for potential bias associated with speed values determined using GPS telemetry data.

## Graphical abstract

We tested the reliability of the Random Encounter Model, a camera‐trap method, to estimate black bear densities in the same study area where we were conducting a hair‐snagging sampling with a traditional spatial capture–recapture model. We also tested the influence of a parameter (average speed) imported from other bear populations that is required to calculate the densities with this camera‐trap estimator. We conclude that the Random Encounter Model can be an affordable alternative to conventional spatial capture–recapture for black bears.

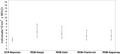

## 1 INTRODUCTION

Estimation of population abundance is an important aspect of many conservation programs, as it provides information on population demography while highlighting the efficiency of conservation actions (Boitani & Fuller, 2000; Gibbs, 2000; Krebs, 2001). In most cases, censuses cannot be conducted, so alternative methods accounting for imperfect detection are used, such as capture–recapture (Efford, 2004, 2011; Schwarz & Seber, 1999; Williams et al., 2002).

Recent developments in capture–recapture modeling have resulted in the ability to account for heterogeneity in capture probability and edge effects (assumption of geographic closure), thereby overcoming two major limitations of conventional capture–recapture estimators (Efford, 2011; Karanth et al., 2006; Kendall et al., 2008). In light of these advantages, spatial capture–recapture models (hereafter SCR) have become largely used to assess the population abundance of many mammals including dingo *Canis familiaris* and red fox *Vulpes vulpes* (Forsyth et al., 2019), wolverine *Gulo gulo* (Royle et al., 2011), grizzly bear *Ursus arctos* (Boulanger et al., 2004; Morehouse & Boyce, 2016), and black bear *U*. *americanus* (Frary et al., 2011; Gardner et al., 2010; Royle et al., 2013). SCR requires individual recognition, often achieved via direct (*e.g*., tag, collar, which requires capturing individuals) or indirect marking (*e.g*., DNA‐based mark–recapture based on hair snagging; see Boulanger et al., 2004; Morehouse & Boyce, 2016). These expensive methods are essential, but can represent important limitations for projects with relatively little financial support. Moreover, marking individuals can be challenging when the studied species is rare or cryptic and difficult to capture (Foster & Harmsen, 2012).

To overcome these limitations, some innovations were proposed in the last decades to avoid the expensive step of capturing and marking individuals. Such innovations include automated camera traps, originally used for species displaying natural marks (*e.g*., stripes) that enable individual identification (e.g., Karanth, 1995). Recently, methods have been developed to estimate density without needing to differentiate individuals, permitting the use of camera traps to estimate the density of species without natural marks. The random encounter model (hereafter REM) estimates the density of a target population using trapping rate, the average size of the detection zone, and knowledge of average movement speed (Rowcliffe et al., 2008). It has been tested on a variety of species (Caravaggi et al., 2016), ranging from small, slow‐moving mammal species (*e.g*., pine marten *Martes martes*; 0.037 km/h; Caravaggi et al., 2016) to medium (*e.g*., European wildcat *Felis silvestris silvestris*; 0.094 km/h; Anile et al., 2014) and large, fast‐moving mammals (*e.g*., lion *Panthera* *leo*; 0.126–0.307 km/h; Cusack et al., 2015). However, many research articles have highlighted the need to validate REM by comparing it to more traditional density estimators and to test its reliability, accuracy, and sensitivity (Kelly, 2008; Lucas et al., 2015; Dénes et al., 2015; Sollmann et al., 2013).

Considering that this novel density estimator uses an estimation of average speed that is not obtained through camera‐trap sampling, its reliability can be challenged if the estimates are greatly influenced by the values used to parameterize them. Although it could be possible to collar animals in the targeted population to obtain this information, doing so would complicate the use of the REM as it requires coupling camera‐trap sampling to telemetry monitoring, thus going against the goal of reducing costs. In some cases, it is possible to set those parameters by relying on information gathered in previous studies conducted on the same species from another population (see Cusack et al., 2015; Zero et al., 2013). However, when this information is not available, many researchers will assume that the use of data from studies carried out on the same species will give a reliable density estimate (see for examples Anile et al., 2014; Balestrieri et al., 2016; Caravaggi et al., 2016; Manzo et al., 2011). As setting an average speed using values originating from other populations is still common, and considering the risk of inducing a bias by doing so, it appears important to evaluate the accuracy and sensitivity of the REM when using parameters taken from other populations.

In this paper, we aim to (i) determine whether density estimation based on REM and camera trapping can replace SCR applied on DNA‐based hair‐snagging surveys by yielding similar estimates and precision levels, at a comparable cost and with a similar effort, and to (ii) characterize the sensitivity of the REM density estimator when using average speed originating from other populations of the same species. We hypothesized that both estimates are comparable in size and precision, but that the REM density estimator is sensitive to the average speed used to parameterize the model. Because SCR models using DNA‐based hair snagging are more commonly used now to estimate bear densities in North America (e.g., Boulanger et al., 2004; Roy et al., 2012; Dussault et al., 2014), we tested these hypotheses using a black bear population found in a study area where the high bear density has led to depredation problems. We first estimated the reference density of a black bear population using a SCR approach with DNA‐based hair snagging. In parallel, a camera‐trap design was set to estimate black bear density using REM, for which we set average speed using telemetry data collected from four different bear populations.

## 2 METHODS

### 2.1 Study area

**FIGURE 1:**
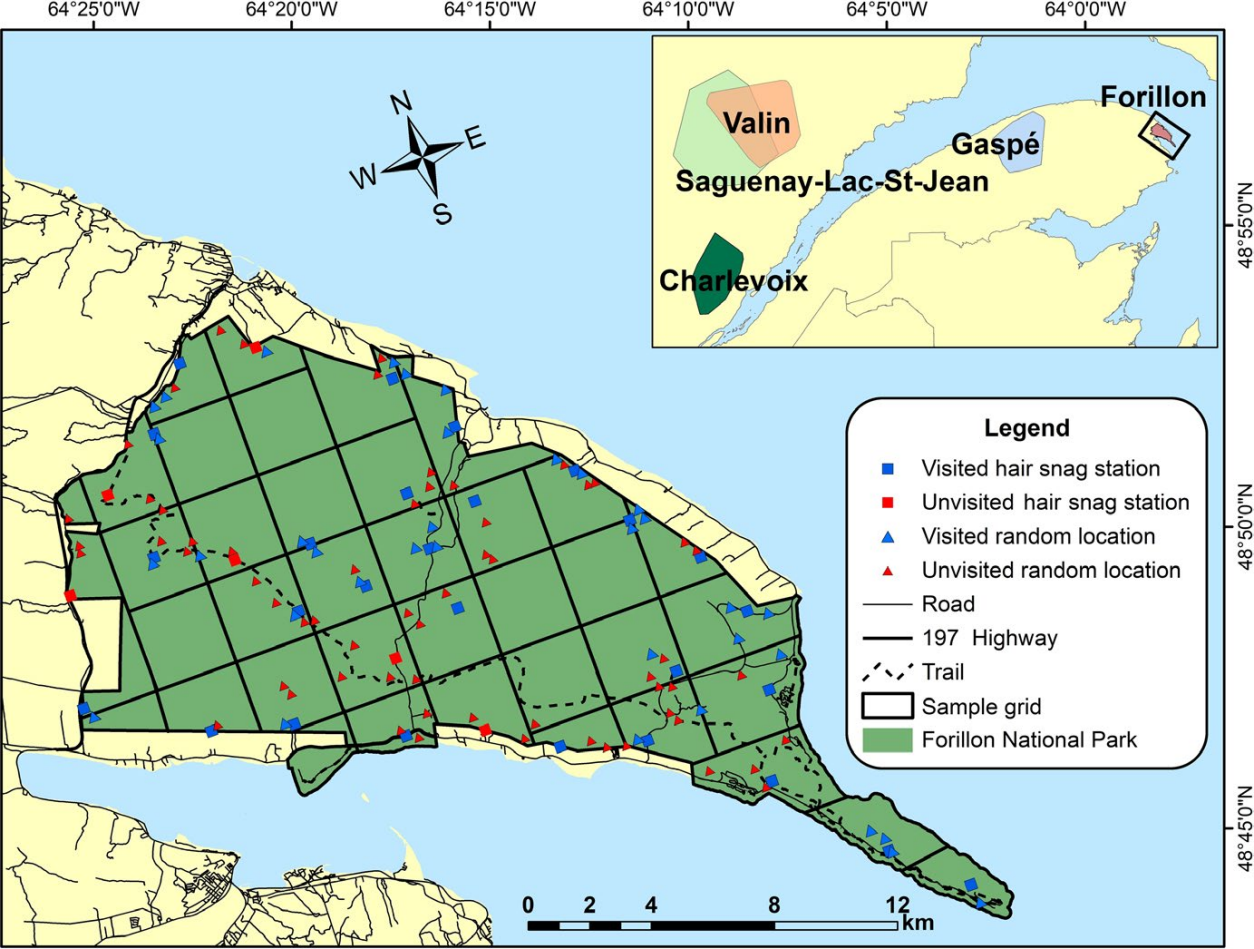
Location of the Forillon National Park and of our study area (Gaspésie Peninsula, Québec, Canada). Upper‐right insert: location of the 4 populations used to set average speed for REM

The Forillon National Park (48° 53’ 45’’N, 64° 21’ 43’’W), located in eastern Québec, Canada (Figure 1), is a relatively small park (*i.e*., 244 km^2^). Found in the eastern balsam fir‐yellow birch bioclimatic domain, the park is characterized by a humid continental climate and a rolling‐hill topography with an elevation ranging between 0 and 450 m asl. The Forillon National Park is delimited by important natural and anthropogenic barriers. Indeed, approximately 80% of the Forillon Peninsula is surrounded by water (north and east: Gulf of St. Lawrence; south: Gaspé Bay) and the remaining 20% is bordered by the 197 highway, where residential development and traffic are significant (4,250 vehicles/day on average, MTQ, 2014) and could act as a relatively nonpermeable barrier. These particularities suggest that the black bear population in Forillon National Park could be geographically closed, at least more so than other bear populations studied in continuous forests. For these reasons, the Forillon black bear population is well suited to compare REM and SCR modeling approaches and to test the sensitivity and reliability of REM models using average speeds of black bears obtained from different populations.

### 2.2 Reference density estimated using DNA‐based spatial capture–recapture

We estimated a “reference density” to compare with the different density estimates obtained via the REM camera‐trap density estimator using a hair‐snag genotyping survey and a DNA‐based capture–recapture statistical method (Woods et al., 1999). We divided the study area into 37 irregular cells of ~7.5 km^2^ and placed a hair‐snag station as close as possible to the center of 33 of these cells; the four remaining cells were discarded due to their inaccessibility and steep slopes (Figure 1). Following Roy et al. (2012), cell size was based on the average home‐range size of female bears, which reaches 10.77 ± 7.06 km^2^ in our study area (Leblanc & Huot, 2000). As the home‐range size of male black bears is larger than that of females (Koehler & Pierce, 2003; Nilsen et al., 2005), we assumed that a 7.5‐km^2^ cell size would provide every female potential access to more than one station within their individual home range and would allow males to access several stations in their home range, maximizing the number of recaptures needed for the spatial capture–recapture approach (Efford et al., 2009).

Hair‐snag stations were composed of two barbwires fixed on tree trunks 35 cm and 65 cm aboveground to delineate an enclosure of 5 × 5 m. Scent lures were used to attract bears in the hair‐snag stations in order to increase the probability of “capture” (Chicoine, 2014). Three types of scent lures were used: A mustelid lure in a pierced plastic bottle was hung in the air at the center of the station, while 100 ml of seal oil was applied to a woodpile in the middle of the station (on the ground), and a 1:1 mix of vegetable oil and anise oil was sprayed on tree trunks found within the barbwire section of the station. Lures were refreshed every week. Stations were sampled for 5 weeks, from 6 July to 18 August 2015, as black bears usually increase their movement rate at this time (Chicoine, 2014), increasing the probability of recapture and consequently yielding more precise density estimates. Stations were visited weekly, and each hair sample was collected individually (*i.e*., multiple hairs tied on a single barb were considered one sample) and identified to record its location on the barbwire (lower versus. upper barbwire, and barb ID‐#; Kendall et al., 2009). This location was used to facilitate the “Mowat 1‐in‐3” subsampling method (see below) as bears left hairs on adjacent barbs when entering in the station (Chicoine, 2014). After each visit, the barbwires were burned using a blowtorch to prevent DNA contamination between sessions (Kendall et al., 2009). Each hair sample was stored in a paper envelope with silica desiccant and freeze‐dried for 24 hr (Kendall et al., 2009, Dussault et al., 2014).

All samples were sent to Wildlife Genetic International (hereafter WGI) for genotyping. Hair samples were subselected based on the “Mowat 1‐in‐3” method, developed by WGI (Mowat et al., 2005); this method offers a good compromise between genotyping many samples that belong to the same individual and missing genetic material from other individuals. DNA was extracted from the samples using QIAGEN DNeasy tissue kits, and the genotyping followed a standard three‐phase approach (*i.e*., first pass, cleanup, and error‐check; Paetkau, 2003, 2004), using seven microsatellite markers (G10L, G10H, UarMU23, UarMU50, MSUT‐2, and G10X) plus ZFX/ZFY for gender.

We analyzed these data using a Bayesian SCR estimator that can be used with data gathered in populations that are not geographically closed (Royle et al., 2009). We have chosen a Bayesian approach rather than a frequentist (i.e., maximum likelihood) method due to our relatively small sample size, as a Bayesian analytical framework does not rely on asymptotic assumptions and the resulting inferences are considered valid for any sample size (Kéry et al., 2010; Link & Barker, 2010; Royle et al., 2009). SCR used different information: the spatial deployment of hair‐snagging traps, capture history, and the characteristics of the state space (*i.e*., the systematic distribution of points covering the trap area, and extended area surrounding it, representing the potential animal activity center). We set the state space as the trapping area with a 6‐km buffer around it (i.e., 3 times the radius of a 10.77 km^2^ home range). As the study area is surrounded by the sea on three sides (north, east, south) and by a major road on the fourth side (west), we were confident that the buffer was sufficiently wide to include all potential home‐range centroids for the bears found in our study area. We then set the upper limit of the potential abundance in the area of interest (M) to 400 individuals and built 8 different candidate models using different combinations of the covariates known to influence detectability (*g0*), that is, sex, behavioral response to first capture, and variables known to influence the scale of movement distribution (*σ*) (*e.g*., sex). We also accounted for the distance between a trap and an individual's activity center (see Table S1). Starting values for parameters were *σ* = 1, *θ* = 0.75, ln_(α0)_ = −4.01, *β* = 0, *ψ* = 0.5 and *ψ*_sex_ = 0.31, the proportion of male in our population sample. Improper priors were used for *α*_0_ and *β* parameters, (0, ∞) for σ, (0.5, 1) for θ and (0, 1) for both *ψ* and *ψ*_sex_. We fit the 8 candidate models using data augmentation and Monte Carlo Markov Chains (MCMC) in the SCRbayes package (Goldberg et al., 2015) implemented in R (R Core Team, 2016). Models were run in three chains of 400,000 iterations with the first 50,000 burn‐in. We assessed convergence using the Gelman–Rubin diagnostic (Gelman et al., 1996). The convergence criterion was met when the value of the Ȓ statistic was <1.1 (Gelman et al., 1996; see Table S3). The density estimate and 95% Bayesian credible intervals were calculated as the mean and the 2.5% and 97.5% quantiles of the posterior distribution of *D* (*i.e*., posterior distribution of abundance parameter divided by the area of the state space). We selected the most parsimonious model by calculating the Bayes factor using the library SCRbayes (Goldberg et al., 2015). The demographic closure assumption was assessed using the test developed by Otis et al. (1978).

### 2.3 Random encounter model (REM)

The REM models population density by describing the contact rate between animals and passive detectors (Lucas et al., 2015), that is, camera traps in our study, without requiring individual identification. The model was developed by Rowcliffe et al. (2008) based on the “ideal gas model” (Hutchinson & Waser, 2007), which estimates the contact rate between gas molecules using the ratio between area covered by molecules and total area. REM relies on three assumptions: (i) animals and cameras conform adequately to the model used to describe the detection process; (ii) photographs represent independent contacts between animals and cameras; and (iii) the population is geographically and demographically closed (Rowcliffe et al. 2008). Rowcliffe et al., (2008) demonstrated that REM is reasonably insensitive to oriented (*i.e*., nonrandom) movements, making the first assumption of REM less important. However, in order to meet the second assumption, particular attention was given to camera placement in order to maintain clear detection zones and avoid bias in trap rates (*e.g*., placing a camera in front of a known animal corridor) (Rowcliffe et al., 2008). This ensured independent contacts between animals and detectors. Finally, we are confident that we met the third assumption, as the geographic and anthropogenic barriers surrounding Forillon National Park are relatively nonpermeable (*i.e*., geographic closure) and the survey period was relatively short (< 10 weeks; demographic closure), and timed after the birth of the cubs and before the sport hunting and trapping seasons (Mowat & Strobeck, 2000). Rowcliffe et al. (2008) adapted the model to estimate density in a given area (i.e., a fraction of a circle) using the following equation:

(1) $$\text{Density}=\frac{y}{t}*\frac{\mathrm{Pi}}{vr\left(2+\theta \right)}$$

where *y* is the number of events (i.e., photographs), *t* the total effort, *v* the average speed of the target population, and *r* and *θ* refer, respectively, to the mean radius and angle of the camera detection zone.

The encounter rate $\left(\frac{y}{t}\right)$, where *y* is the number of events and *t* the total effort, was assessed using 47 remote cameras (Spypoint model I‐6: *n* = 13 and model Tiny: *n* = 22; Reconyx model RM45: *n* = 4; Moultrie model A‐7i: *n* = 8). All cameras used passive infrared and movement sensors, and the trigger sensitivity was set to its maximum. The cameras were programmed to take three consecutive pictures when triggered and were randomly distributed within the 7.5‐km^2^ sampling cells by generating 10 random points in each cell; one to five of these points were then selected based on their accessibility (on foot or with an ATV). The average distance between two adjacent cameras was 0.672 km (ranging from 0.223 to 3.304 km).

The radius (*r*) of the camera detection zone was assessed through several trials during which a person crossed the camera detection zone perpendicularly. The detection arc (*θ*) of the camera detection zone was assumed to be the value found in the specifications of each camera model.

At microsite scale, the cameras were installed in a direction that provides a relatively clear detection zone, without being faced deliberately toward a trail used by wildlife. We removed branches and stems in front of the cameras to maintain consistency across cameras regarding the detection zone area and to reduce the number of empty pictures triggered by branch movements that trigger the sensors. At each selected point, a camera was fixed on a tree 75 cm aboveground for a minimum of 21 days, leading to the placement of 110 cameras from 1 July to 9 September 2015. When a bear remained in a camera's detection zone for a period of time, all the pictures taken during that time were considered to be a single event (Cusack et al., 2015). As suggested by Keim et al. (2019), we considered as two separate events instances when a bear triggered a camera, left the monitoring station, and returned to the same detection zone >10 min later.

The average speed (i.e., movement rate, measured as the Euclidian distance between two successive relocations divided by the time elapsed between them) was not available for the study population (Forillon), so we used GPS telemetry data gathered from four different black bear populations in the province of Québec during the last 19 years (see Table 1 for more details). For each dataset, we calculated average speed (*v*) and its standard error using only the relocations collected from July to September (i.e., the same period during which of our camera‐trap and hair‐snag data were collected) with a minimum fix rate of 8 locations/day (*i.e*., 1 location every 3 hr) to reduce bias in the estimation of average speed (Rowcliffe et al., 2008).

**TABLE 1:** Characteristics of the five telemetry studies conducted on black bear populations that were used to set parameters for REM (average speed)

	Location			
	Gaspésie	Charlevoix	Saguenay‐Lac‐St‐Jean	Valin
No. of bears collared	19	13	21	59
Mean no. of locations per home range	752	494	2,507	1,117
No. of locations to calculate average speed	3,063	1,567	21,867	26,094
Survey duration	2003–2004	2005–2006	2008–2010	2011–2012
Number of Males versus. Females	N/A	6 versus 6	18 versus 0	12 versus 16
Average speed (in km/h) ± *SD*	0.233 ± 0.315	0.309 ± 0.584	0.309 ± 0.449	0.258 ± 0.306
Reference	Mosnier et al. (2008)	Leblond et al. (2016)	Massé et al. (2014)	Lesmerises et al. (2015)

Abbreviation: N/A, information not available.

We assessed the variance of REM density estimates using the delta method, which approximates the variance of any parameter that is a function of random parameters that have their own variance estimation (Powell, 2007; Seber, 1982). In this case, the method incorporates trap‐rate variance, evaluated using nonparametric bootstrapping and resampling (350,000 iterations of camera location replacement), and the standard error of movement rates, *r* and *θ,* allowing the inclusion of variation in detection zone parameters due to the use of different camera‐trap models (Rowcliffe et al., 2008; Zero et al., 2013). The confidence intervals of REM models thus correspond to the 2.5% and 97.5% quantiles of the nonparametric bootstrap.

In order to compare the applicability of the two methods, we calculated the costs (material, DNA analyses) and the effort spent in the field to deploy these two different sampling designs and collect data, while assuming that the time spent conducting statistical analyses was similar for SCR and REM.

### 2.4 Influence of imported parameters on REM sensitivity

We explored the potential impact of variation in the average speed parameters used to calibrate the model on REM sensitivity. We first assessed the interpopulation and intrapopulation variation in speed (i.e., movement rate) between the four telemetry datasets using a factorial (type III) ANOVA with population ID as a factor. A log transformation was applied on the speed parameter so that the normality of residuals and variance equality assumptions were met; these assumptions were verified using visual inspection of residuals. The percentage of the total sum of squares attributed to the factor was considered to be the interpopulation variation, and the percentage of the total sum of squares attributed to residuals was considered to be the intrapopulation variation. If a greater part of the explained variance is associated with interpopulation variability in speed, it would mean that there is a greater risk of estimate bias following the importation of a speed parameter from a different population than the one studied.

Variability in speed can be low if movement is essentially constrained by morphological factors (Seyfarth et al., 2002) rather than on ecological factors (e.g., Doherty et al., 2019). We illustrated the influence of a small variation in average speed on REM density estimation for two contrasted velocities: one with a low movement rate (0.10 km/h) and one with a higher movement rate (1 km/h), for which we induced an absolute increase of speed of 0.015 km/h. By doing so, we aimed to describe the influence of a small (but plausible) underestimation or overestimation of the average speed on the resulting density estimate and determine for which type of species (slow‐moving or fast‐moving species) this potential bias could be more important.

## 3 RESULTS

### 3.1 DNA‐based spatial capture–recapture

We recorded at least one black bear visit for 27 out of our 33 hair‐snagging stations. Visited stations collected hairs from 5.7 bears (on average, ranging from 1 to 18 different individuals) during the sampling period (5 weeks). A total of 1,023 hair samples collected in our hair‐snag stations were sent to WGI for genotyping. WGI excluded 36% of the samples (*n* = 373) based on their subselection rules (*i.e*., Mowat 1‐in‐3) and excluded another 20% (*n* = 200) that lacked suitable DNA material. In the remaining samples, 5% (*n* = 50) did not look like bear hair, the genotyping of 6% (*n* = 64) failed and 0.2% (*n* = 2) had mixed results, leading to 334 successful samples assigned to 72 black bears (23 M : 49F). The recapture rate reached 77%, with 34 of the 72 individuals recaptured only once while some bears were recaptured up to 5 times.

**FIGURE 2:**
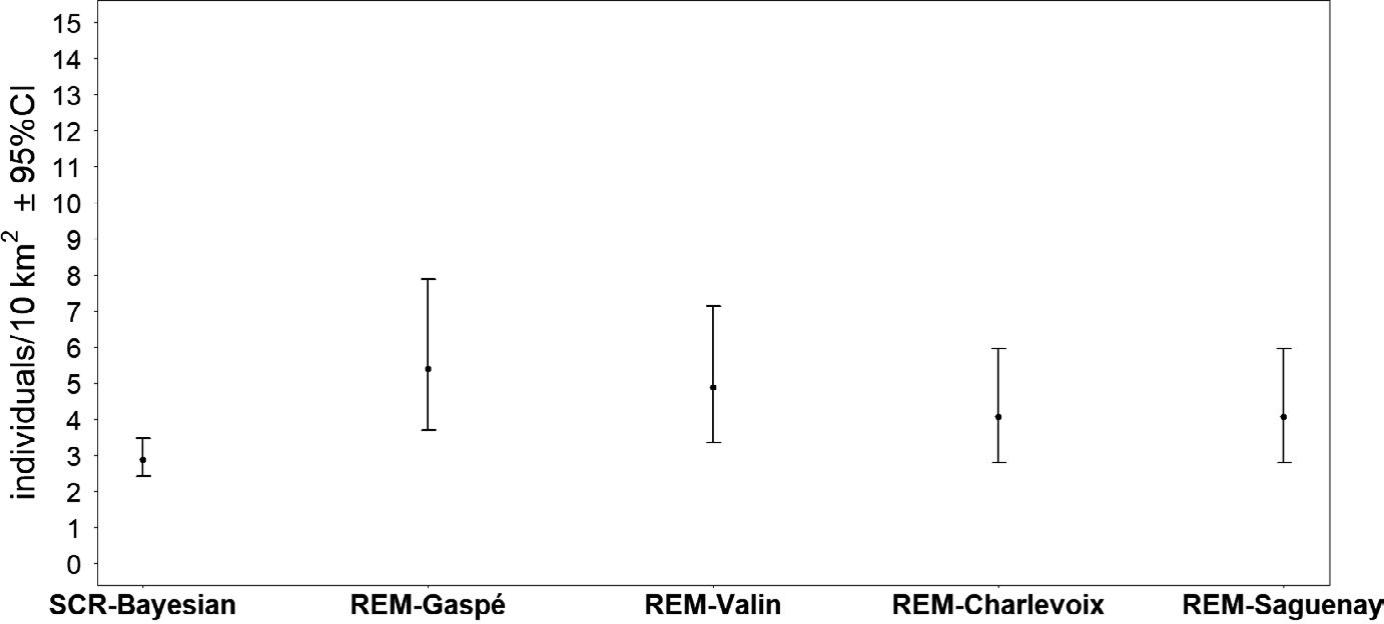
Comparison of the density estimates (±95%CI) obtained from hair‐snag stations (as a reference) and the camera‐trap REM model using different speed values (populations). SCR‐Bayesian = spatial capture–recapture model ranked using the package SCRbayes (Bayesian; mean, 95% credible interval). REM‐Gaspé = Random encounter model (mean, 95% confidence interval) parameterized with the average speed value from the Gaspé dataset, REM‐Valin = Random encounter model (mean, 95% confidence interval) parameterized with the average speed value from the Valin dataset, REM‐Charlevoix = Random encounter model (mean, 95% confidence interval) parameterized with the average speed value from the Charlevoix dataset, REM‐Saguenay = Random encounter model (mean, 95% confidence interval) parameterized with the average speed value from the Saguenay dataset

Our study population met the demographic closure assumption according to the Otis closure test (Z = 0.33, *p* = .63). All Bayesian candidate models of spatial capture–recapture converged according to Gelman–Rubin diagnostics. The most parsimonious model according to the Bayes factor was Model 1 (Table 2), where sigma (*σ*) = 0.74 (mean) ± 0.04 (*SD*) and *g0* = 0.59 ± 0.12 (*SD*) were considered to be constant, yielding a density of 2.87 bears/10 km^2^ (95% CI [2.41–3.45]; Figure 2).

**TABLE 2:** Estimates of black bear density (no. of individuals/10km^2^) in Forillon National Park (Québec, Canada) in 2015 following comparison of SCR models with Bayesian approach. Models are described in Table S1 and are ranked using the Bayes Factor, where a greater value represents the most parsimonious model. Confidence intervals are shown with the 95% confidence interval (95% CI; [lower : upper]) and the coefficient of variation (CV)

Model	Composition	Bayes Factor	Density	95% CI	CV(%)
1	*g(.)s(.)*	1.00	2.87	[2.41:3.45]	18
3	*g(.)s(S)*	<0.001	3.24	[2.62:4.06]	22
2	*g(S)s(.)*	<0.001	2.93	[2.43:3.54]	19
6	*g(bS)s(.)*	<0.001	3.83	[2.93:5.12]	29
8	*g(bS)s(S)*	<0.001	4.14	[3.07:5.85]	34
4	*g(S)s(S)*	<0.001	3.16	[2.60:3.87]	20
5	*g(b)s(.)*	<0.001	3.75	[2.88:4.98]	28
7	*g(b)s(S)*	<0.001	4.81	[3.35:7.01]	38

The material used to carry out the hair‐snagging fieldwork operations had cost 1,450 CAD and necessitated 672 person‐hours (deployment and visiting of the sampling design) while the genetic analyses had cost 20,000 CAD.

### 3.2 Random encounter model (REM)

Based on in situ trials and camera specification, the camera detection zones had a mean radius of 11.6 ± 0.63 m and a mean arc of 0.698 ± 0.07 radians. Some logistic issues (*e.g*., lack of batteries or full memory cards) have compromised the complete deployment of the cameras in all the sampling stations, so our effort reached 2,236 camera‐days in 99 locations (sites) with a mean of 378 (±633, *SD*) pictures per location. We thus captured 67 independent black bear events in 36 different locations. Nonparametric bootstrapping led to a mean trap rate of 2.88 (±0.48, *SD*) event/100 camera‐day. Based on the REM, the black bear population density ranged from 4.06 to 5.38 individuals/10km^2^ (Figure 2) with a mean coefficient of variation of 39%. The camera‐trapping material cost 16,813 CAD; the deployment, visit, and relocation of our camera‐trap stations required 448 person‐hours while the analysis of the photographs involved 75 person‐hours.

### 3.3 Influence of model imported parameters on REM sensitivity

The factorial ANOVA showed that the average speed, needed to parameterize the REM, differed between the bear populations we considered (*F*_(3,52,585)_ = 34.59, *p* < .001). The sum of squares revealed that the “population” factor (*i.e*., interpopulation variability) explained only 0.2% of the variance in speed, while the variation within a given population (*i.e*., intrapopulation variability) explained 99.8%. Despite the statistical differences in average speed between populations, the speed values were more different between (and within) individuals in a population than between populations.

**FIGURE 3:**
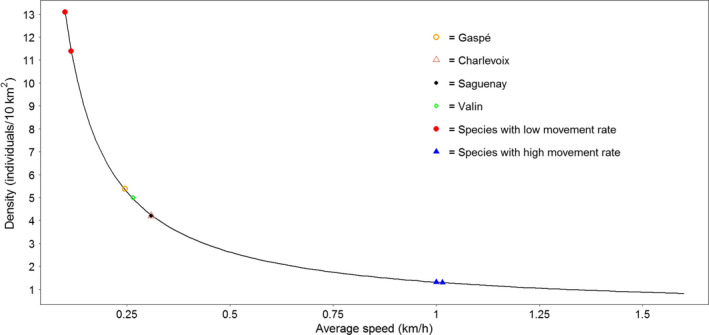
Variation of mean density estimate in response to variation in average speed (solid line). Points represent the different REM density estimates for the black bear population of Forillon National Park in 2015. Red circles represent the results of a variation of 0.015 km/h on density for a simulated species with an average speed of 0.1 km/h. Blue triangles represent the results of a variation of 0.015 km/h on density for a simulated species with an average speed of 1 km/h

We highlighted a negative exponential relationship between estimated density and average speed (movement rate; Figure 3), indicating that variation of speed has more impact on the estimated density for species with a low movement rate, thus suggesting that REM sensitivity to variations in speed is asymmetrical. Based on the structure of the REM equation, an absolute increase of 0.015 km/h in speed for species with a low movement rate (*i.e*., 0.10 km/h) induced a decrease in density of 2 individuals/10 km^2^ in our study system (Figure 3). Conversely, an increase of 0.015 km/h in speed for a species with a higher movement rate (i.e., 1.00 km/h, than ~2%) resulted in a decrease of the estimated density of only 0.02 individuals/10km^2^ (Figure 3), a variation level 100 times lower for the same absolute change in speed.

## 4 DISCUSSION

### 4.1 Comparison of the density estimates between REM and DNA‐based SCR

Our study aimed first to compare the bear density estimates obtained from a camera‐trap method with an estimate obtained in the same study area using the more “traditional” spatial capture–recapture (SCR) model. The “reference” density we obtained using a Bayesian SCR approach yielded a lower density estimate, albeit not statistically different (*i.e*., the 95%CI of REM overlap those of SCR), than those computed using the REM for 3 of the 4 GPS telemetry datasets we had. The only exception was for the REM model parameterized with the average speed calculated with the Gaspé GPS telemetry dataset, which led to a significantly higher density estimate than the one obtained from the SCR. Although we do not have insights regarding the accuracy of each of these estimates versus. the “real” density of bears in the Forillon National Park, the fact that density estimates did not differ between both methods is reassuring and suggests that REM can be an efficient alternative to more invasive spatial capture–recapture methods.

Nevertheless, density estimates obtained with the REM estimator were slightly higher than the reference (DNA‐based SCR) estimate, suggesting a potential bias toward density overestimation with REM. We believe that this difference could be related to the subtle bias in movement rate (i.e., as the proxy of speed) calculations induced by the time lapse between successive GPS collar relocations. Indeed, the distance covered by an animal is assumed to be a straight line between successive relocations, so a greater time lapse between GPS relocations will result in an increase of unobserved tortuosity. Ultimately, this will lead to an underestimation of movement rate, and of the average speed value used, resulting in a potential density overestimation (Rowcliffe et al., 2012). This phenomenon was described by Pépin et al. (2004) and Mills et al. (2006), who respectively found that for wolves (*Canis lycaon*) and red deer (*Cervus elaphus*), a 2‐hr fix rate between telemetry relocations led to a ~ 60% underestimation of the daily distance traveled by animals. In our study, most of the relocations used to approximate the speed parameter had a 2‐hr fix rate, suggesting that this parameter might be underestimated and that, consequently, our density estimates could have been overestimated. When a correction factor is applied on black bear movement rates to account for the 60% underestimation (according to Pépin et al., 2004 and Mills et al., 2006), the estimated black bear population density ranged from 2.86 to 3.75 individuals/10km^2^, which is closer to the Bayesian spatial capture–recapture estimate of 2.87 individuals/10km^2^. This highlights a potential improvement for REM when the speed parameter is inferred from telemetry, but it still needs to be tested. In other words, the uncertainty associated with this correction factor and its influence on the precision of the density estimate need to be precisely calculated, a task that will require further research efforts. Nevertheless, our results indicate that with some improvements, the REM could represent an interesting alternative to more traditional capture–recapture methods applicable to large mammals.

Regarding estimate precision, the Bayesian SCR method produced a more precise estimate than those obtained with the REM; this is possibly related to the small number of bear pictures in our camera‐trap dataset. Indeed, as in all capture–recapture calculation methods, the precision of density estimates is strongly related to the number of “recapture” events (see Krebs, 1999; Seber, 1982). As suggested by Rowcliffe et al. (2008), increasing the number of events via an increase in the number of camera‐trap locations as well as effort by location (i.e., number of days each camera is in action) would have narrowed the width of the confidence interval, resulting in more precise estimates. A purely random distribution of the camera‐trap stations could also slightly influence the estimate, albeit an ongoing companion study suggests that this potential source of bias is quite small for large mammals (P. Pettigrew, F. Lesmerises and M.‐H. St‐Laurent, *unpublished data*).

The comparison of the costs and investment of the two methods suggests that the deployment of the REM sampling design was less expensive and required lower effort (number of person‐hours) than the DNA‐based hair‐snagging SCR design. One of the main advantage of the REM approach is the possibility to conduct recurrent camera‐trap surveys using the same cameras, at a lower cost. If GPS data are available for several species, it could also be possible to conduct multispecies density surveys with the same camera‐trap network by defining station densities based on species having the smallest home‐range size. Therefore, we recommend using camera trapping and the REM estimator over DNA‐based hair‐snagging SCR when budget is limited and recurrent density estimates are needed. However, if the study objectives require demographic and genetic informations (e.g., sex‐ratio, genetic diversity) besides density estimation, DNA‐based hair‐snagging SCR could be more relevant.

### 4.2 REM sensitivity to imported speed values

Our study also aimed to assess the sensitivity of REM to the use of calibration parameters originating from other bear populations. We consider that the risk of biasing the estimate density by using an average speed value imported from a companion study exists, but is limited, as suggested by the low variability in average speed between our four GPS datasets. This resulted in four REM density estimates that were not statistically different from each other. This latter part of the explanation is nevertheless debatable when looking at the confidence interval sizes and overlaps of the four REM estimates.

The REM’s sensitivity to changes in the absolute value of speed is undisputedly real, as shown by the negative exponential relationship linking estimated density and average speed values. This result highlights the consequences of using a biased or unrealistic average speed (or movement rate), especially for species with low movement rates. This is not trivial, considering that the average speed of studied species in many published applications of the REM was similar to our example of a slow‐moving species (*e.g*., 0.07 km/h in Rovero & Marshall, 2009; 0.09 km/h in Anile et al., 2014; 0.04km/h in Caravaggi et al., 2016), which was more impacted by subtle variations in average speed values than fast‐moving species. In our study, using average speed values from different populations did not have an important effect on black bear density estimates, as the very low interpopulation variation in average speed and the relatively high average speed of bears (0.279 km/h) limited the consequences of variation in speed on the resulting estimates. Average speeds calculated from GPS data obtained from different black bear populations in Québec were relatively similar despite the fact that these bears were living in quite different environments yielding different availabilities and diversities of food resources (M.‐H. St‐Laurent, *unpublished data*). Based on these observations, we recommend calculating the average speed value using GPS relocation telemetry data from the studied population, preferably scheduled with a high fix rate. If not possible, we consider that it is more appropriate to use average speed (or movement rate) values available in the literature for different populations of the studied species that live in similar environments than to apply an arbitrary value from only one population. However, this would require to test the influence of these different values on the resulting REM density estimates. Being aware of the potential underestimation of average speed related to GPS telemetry and estimating the average speed on a large sample size of collared animals are essential to estimate efficiently the within‐individual variation in movement rate. Although we have not tested this precisely, our results suggest that it could be risky to import an average speed value from another species, even a taxonomically related one (e.g., in our case, grizzly bear).

## 5 CONCLUSIONS

Our study suggests that the Random Encounter Model, a camera‐trap density estimator, could be an interesting alternative to conventional spatial capture–recapture estimators as it involves lower cost and effort while yielding a potentially reliable and accurate density estimate. However, caution is required to avoid some pitfalls associated with sampling effort (i.e., the density of camera‐trap stations) and the estimation of an accurate average speed value for the studied species, especially when it comes to slow‐moving species. Our results suggest that it is safer to derive the speed parameter from more than one population when using information from other populations to calibrate the REM model. The bias in speed parameter induced by the time lapse between GPS relocations seems to be a driver of density overestimation when using REM, but using a correction factor appears to be a suitable solution to reduce this bias, although further research is required to efficiently integrate the uncertainty associated with this correction factor. While our SCR analytical method considered the influence of behavioral and individual effects, we recognize that the REM model did not allow us to consider such sources of confounding variation per se. This limit should be considered when direct comparisons are made between estimates obtained from spatial capture–recapture and estimates coming from this camera‐trap density estimator. Although there is still some room for improvement, we are confident that using camera traps for density estimation could be an affordable and reliable tool to monitor density and that such methods could diversify the toolbox of wildlife biologists.

## CONFLICT OF INTEREST

The authors declare that there is no conflict of interest.

## AUTHOR CONTRIBUTION

**Pascal Pettigrew:** Conceptualization (equal); Formal analysis (lead); Investigation (lead); Methodology (equal); Writing‐original draft (lead). **Daniel Sigouin:** Conceptualization (equal); Funding acquisition (lead); Investigation (supporting); Project administration (equal); Resources (equal); Supervision (supporting); Writing‐review & editing (equal). **Martin‐Hugues St‐Laurent:** Conceptualization (equal); Data curation (lead); Formal analysis (supporting); Funding acquisition (supporting); Methodology (supporting); Project administration (equal); Resources (equal); Supervision (lead); Visualization (lead); Writing‐original draft (supporting); Writing‐review & editing (lead).

## Supporting information

Table S1

## Data Availability

Data are archived on DRYAD: https://doi.org/10.5061/dryad.g1jwstqqx.
