# Supplementary material for: Testing the precision and sensitivity of density estimates obtained with a camera‐trap method revealed limitations and opportunities
Source: Ecol Evol. 2021 May 7;11(12):7879–89. doi: 10.1002/ece3.7619 (PMC8216954; doi:10.1002/ece3.7619)
Supplement: Supplementary file 1 — Table S1 [file ECE3-11-7879-s001.docx]

**SUPPORTING INFORMATION**

Pettigrew, P., D. Sigouin and M.-H. St-Laurent. Testing the precision and sensitivity of density estimates obtained with a camera-trap method revealed limitations and opportunities. Ecology and Evolution.

**Table S1.** Candidate models used to estimate density with spatial capture-recapture using the Bayesian approach, where g0 = detection probability when the home-range center and hair-snag station coincide, and *σ* = spatial range over which the detection function is decreasing when the distance to hair-snag station increases.

| **Model** | **Composition** | **g0** | *σ* |
| --- | --- | --- | --- |
| 1 | *g(.)s(.)* | Constant | Constant |
| 2 | *g(S)s(.)* | Influenced by sex | Constant |
| 3 | *g(.)s(S)* | Constant | Influenced by sex |
| 4 | *g(S)s(S)* | Influenced by sex | Influenced by sex |
| 5 | *g(b)s(.)* | Influenced by behavioral response to first capture | Constant |
| 6 | *g(bS)s(.)* | Influenced by behavioral response to first capture and sex | Constant |
| 7 | *g(b)s(S)* | Influenced by behavioral response to first capture | Influenced by sex |
| 8 | *g(bS)s(S)* | Influenced by behavioral response to first capture and sex | Influenced by sex |
